# Selections and Abstracts

**Published:** 1912-10

**Authors:** 


### SELECTIONS AND ABSTRACTS

### AMERICAN PROCTOLOGIC SOCIETY
### FOURTEENTH ANNUAL MEETING, HELD AT ATLANTIC CITL, N. J., JUNE 3 and 4, 1912.

*(Continued from Last Issue.)*

*Acute Post-Operative Intestinal Paresis.—By J. A. MacMillan, M. D., of Detroit, Mich.*

1. Definition:—A paralysis of a portion of the intestine which suddenly dilates and becomes the receptacle for gas and fecal matter.

2. Etiology:—Not known, but probably due to sepsis, trauma, etc.

3. The lesion is probably in the sympathetic nervous system.

4. The treatment consists of gastric lavage, enemata, and enterostomy.

5. Precautions attending a secondary operation.

---

*Prophylaxis and Treatment of Post-Operative Retention of Urine.—By Frank C. Yeomans, M. D., of New York City, N. Y.*

Ascertain and correct, if possible, lesions of the urethra and bladder in advance of operation.

Physiology of urination. Factors that interfere with it after operation.

Prophylaxis.—Urinary antiseptics and posture.

Treatment.—Suggestion, local applications, medicine, standing. Aseptic catheterization.

---

*Intra-Rectal Rupture of Suppurating Sinus from Hip-Joint Disease.—By Ralph W. Jackson, M. D., of Fall River, Mass.*

To meet the difficult problems presented by an unusual case, involving the rupture, internally, into the rectum, and externally, near the anus, of a sinus from a tubercular hip, the writer has sought by radiographic study, research of literature, and correspondence with proctologic and orthopedic authorities, information as to frequency, pathology and operative possibilities, of such cases; and with the following conclusions:

1. That intra-anal or rectal rupture of a coxitic sinus occurs rarely, but not with extreme infrequency.

2. That such opening, involving probably considerable mixed infection of the joint beyond what would occur if the opening were external.

3. That likewise tubercular infection of the rectum might arise.

4. That intra-anal opening is quite easily treated and much of the mutual risk of infection removed.

5. That intra-rectal opening is in most cases (unless the sinus approaches from low down) too high to turn aside in any way and give an external discharge and consequently the risk must continue.

6. That operating for such purpose is likely to create at once a complete rectal fistula where none existed before, because of the surgical difficulties in the way of securing permanent closure of the internal opening.

7. That it is a very rare and most unfortunate occurrence for such an abscess to point both externally and internally; an external incision should be made, if sure that internal rupture has not occurred; but avoided, if possible, if it has occurred, because of the fistula thereby created.

8. That whatever the etiology, such a fistula is a particularly troublesome one, and the wisdom of trying to better it surgically is fairly debatable ground.

He stated that while the rectum is easily inspected by various

*Preliminary Report of Two Cases: (a) Keloidal Tuberculoma; (b) Fibromatous Keloid.—By Alois B. Graham, A. M., M. D., of Indianapolis, Ind.*

The writer presented a brief preliminary report of two exceedingly rare rectal cases. Both of the cases are of interest in that they emphasize the following points:

1. A benign neoplasm, involving the peri-anal, peri-rectal and surrounding structures, may be the end-result of an inflammation of these structures.

2. An inflammation of these structures is due, in a large proportion of cases, to extension from an anal or rectal inflammation.

3. A benign neoplasm may produce a marked deformity of the structures which it involves. (The writer showed photographs of his two cases.)

4. A careful pathologic study is essential for making a correct diagnosis of the neoplasm.

5. A correct diagnosis of the neoplasm can be made, and yet its etiology remains vague.

A history of the cases was given, together with a description of the operations performed. The pathological report shows one case to be Keloidal Tuberculoma; the other, Fibromatous Keloid. The first case insisted upon leaving the hospital and was discharged as improved. The second case, that of Fibromatous Keloid, is still under the observation of the writer. Hence the preliminary report.

In conclusion, the writer stated his object for reporting these two cases. He is firm in his belief that an anal or rectal inflammation was the origin of the diseased conditions presented by these patients. Both cases, therefore, emphasize the necessity for and the importance of rectal examinations. It matters not how slight the ailment may be, a careful inspection of the anus and rectum should be made. If such a rule were followed by every physician and surgeon, such case reports would not be possible.

---

*Some Practical Points Gleaned from the Observations of a Proctologist.—By Samuel T. Earle, M. D., of Baltimore, Md.*

Dr. Earle reported a case of Primary tubercular ulceration of the right buttocks, which was not connected with the rectum by a fistulous tract. In this report it differed from the one reported by him in his work on "Diseases of the Anus, Rectum and Sigmoid," Figure 62, page 201. It was excised by the thermo-cautery knife, after which it healed very promptly.

Dr. Earle also reported a very aggravated case of pruritus ani, which had resisted local applications, autogenous vaccines and treatment by the X-Ray. Under local anaesthesia he found an ulcer over the posterior commissure just above the internal sphincter, which connected on each side with numerous submucous and subcutaneous superficial fistulae which enveloped the entire anal margin and connected with each crypt of Morgagni. The ulcer was incised, the scar tissue at its base removed, and the fistulous tracts were all opened up. There was only an occasional twinge of itching following the operation, and he made a speedy recovery.

---

*The Subnormal Colonic Function as a Diathesis.—By J. Coles Brick, M. D., of Philadelphia, Pa.*

The writer was led to investigate the causes of a persistent case of constipation, which had existed since childhood, and which was of an average duration of 7 days, in a young woman of 18 who was in seemingly good health, but whose father having had the same condition and who had subsequently developed a case of chronic arthritis deformans. The young woman had been treated by many doctors and by many methods, but all without any more than temporary success.

Resort was finally made to X-Ray examination after giving a bismuth meal. The plates showed that at two points, viz: the cecum and the rectum, the colonic contents remained for three days, and operative measures were decided on. No abnormality was found except an old and thickened appendix, containing 3 concretions, and the tip being adherent to the ovary. As there were some moderate sized hemorrhoids present, these were removed at the same time as the appendix, and the patient made a good recovery. The X-Ray plate showed a very moderate degree of visceroptosis, and a "Storm" belt was ordered. The patient has had a regular bowel movement daily, with the use of a mild laxative which it had been impossible to produce at any previous time.

Examination of the X-Ray plates showed a bilateral calcification of the costal cartileges, which the writer thought was an early symptom of arthritis deformans, and after discussing the various theories of the cause of the disease, accepts the theory that it is a toxic trophoneurosis affecting the cerebro-spinal nerves, with its infectious focus in the gastro-intestinal tract.

The essayist believes that all cases of persistent constipation should be examined by all the means at our command, and, finally, not only by the administration of bismuth by the mouth, but by injection, with X-ray examination—conditions requiring operative interference will frequently be found by this means, and corrected surgically.

Arthritis deformans is a most ancient disease, and evidences of many cases are shown to have existed before the pyramids were built, and that it is not only possible but probable that the infection comes from the intestinal tract, and that if the cause is removed early before the destructive changes have occurred these cases can be cured, and even the advanced cases have their progress arrested.

---

*The Three-Step Operation in Tumors of the Sigmoid and Co'on.—By James P. Tuttle, A. M., M. D., of New York City, N. Y.*

Dr. Tuttle described the operation as follows: Incision is made in the outer border of the left rectus. Tumor is brought out on the abdominal wall. Peritoneal layers of the meso-sigmoid are incised well above and below the tumor, and stripped back so as to expose the blood-vessels, fat and glands, which may be in the meso-sigmoid; the latter are stripped toward the intestine until the blood-vessels are bare and the supply to the bowel is easily visible. The sigmoidal artery is tied in two places and cut between and the proximal stump dropped back into the abdominal cavity. The raw surface in the abdomen is covered by suturing the two peritoneal layers of the meso-sigmoid together over the arterial stump. The two legs of the sigmoid are sewed together laterally to make a spur, after the method of Bodine. The peritoneum is sewed around the bowel; the muscles drawn together; the skin wound closed, attaching it to the bowel. In forty-eight hours the tumor is excised by a V-shaped incision. Two days later, the spur is cut away by pressure-forceps. After this is completed a long rectal bougie is passed up through the bowel beyond the artificial anus, in order to press the spur back and obtain a large caliber at the site of the resection. When the wound made by the pressure-forceps is healed, the artificial anus is closed by the extra-peritoneal method of the author.

---

*The X-Rays as an Aid in Making Diagnoses of Conditions in the Rectum and Other Portions of the Large Intestine.—By J. R. Pennington, M. D., of Chicago, Ill.*

He stated that while the rectum is easily inspected by various specula, and the sigmoid is less readily accessible by the use of sigmoidoscopes, such as the one with insufflation devised by him, the colon is inaccessible and its exact position difficult to ascertain. Very often it is also difficult to determine and locate pathologic conditions in the large intestines.

Until recently, the means of diagnosis have been limited to those used in other portions of the alimentary canal, viz: Inspection after dilatation of the bowel with air or water, Palpation, Percussion, and Trans-illumination. All of these are open to the objection that they are uncertain.

The writer observed in the latter part of 1899, that by introducing some agent into the large bowel which would cast a shadow, the X-Rays may become useful in making a diagnosis of conditions in the twin cavities. It is only recently, however, that such procedures have become of practical value.

A bismuth meal is useful in diseases of the stomach or duodenum, the agent being suspended in milk, acacia water, thick soup or some similar vehicle.

But for the large bowel, the action of bismuth per os is very slow. One author estimates that it requires from 12 to 15 hours for the bismuth mixture to reach the ileo-cecal valve; about 24 hours to gain the transverse colon, and 36 hours to penetrate to the sigmoid. By the method advocated this is done, so to speak, instantaneously.

Coming now to the technic: The patient's bowels are first cleansed by means of laxatives and injections. He is then placed in the knee-shoulder position, and from 25 to 30 ounces of the mixture used for casting the shadow injected into the large intestine. For this purpose the author uses an ordinary irrigator and a short rectal tip. A long rectal or colonic tube for administering the injection is unnecessary. After the suspension is injected the patient lies on his right side for a few moments so part of the mestrum may pass into the cecum. He is then placed in either dorsal or ventral position on the radiographic table and the picture taken.

### A Mystery of Ether Anesthesia.

If one ounce of essential oil of orange and three ounces of water are placed in the hot water bottle of Gwathmey's three bot-the vapor anesthesia apparatus, and ether in the other two bottles, not only is the odor of the anethetic completely disguised, so that the patient is unable to tell when its administration begins, but other important results are obtained; the patient passes into surgical unconsciousness without any preliminary stage of excitement, anesthesia is easily maintained with less than half the quantity of ether required by the usual cone method, and the patient quickly and comfortably regains consciousness without vomiting or even nausea, except in very rare instances.

Four operations were performed at the United States Naval Hospital, Brooklyn, on September 9th, on typically robust and not conspicuously temperate service patients, one for hernia, one for double hernia, one for varicose veins, and one for hemorrhoids, the ether being administered according to the foregoing method by its inventor, Dr. James T. Gwathmey. Owing to the somewhat inaccurate reports contained in the newspapers, the facts herein noted were obtained from the surgeons present. In all these cases anesthesia was obtained with a total absence of the unpleasant phenomena and sequelæ. Recovery followed hard upon the completion of each operation, and the patients presented a perfectly normal appearance. Examination of the urine was negative. In one case, that of the hemorrhoids, there was a temporary retention of urine, a common sequel, however, of such operations. The ease with which anesthesia was induced was the occasion of surprise and comment on the part of the attending surgeons of the navy, who saw immediately the great advantages presented by this method for use on board ship, doing away, as it does, with the necessity for the cumbersome gas oxygen apparatus, and cutting the necessary supply of ether in half; two ounces an hour are ample in this method.

Those who have read the inventor's description of his vapor apparatus[2] will recall that it was suggested that oil of bergamot or terpineol be used as a preliminary inhalation. Doctor Gwathmey was accidentally made aware of the singular effect of penetrating odors preliminary to anesthesia by his practice of soothing and reassuring frightened children by putting a few drops of the nearest convenient perfume on the inhaler and inviting them to note the familiar harmless result. Observing the quiet manner in which the little patients subsequently lapsed into unconsciousness, he cast about for an odor of superior penetrating quality. He noted in Tigerstedt's *Physiology* the statement made by Zwaardemaker, after tests with his olfactometer, that essential oil of orange had ten times the penetrating quality of ether vapor, 0.00005 mg. in fact sufficing perceptibly to affect one liter of air. So marked, however, was the calming effect of this oil that Doctor Gwathmey could not dismiss the idea that it had some anesthetic effect of its own, and experiments on guineapigs were required to dispel the illusion. These animals exhibited a philosophic calm that might be construed as an expression of enjoyment, but were not rendered in the least unconscious.

We are confronted here with the problem that has so far eluded solution. The problem of noci and anoci associations finds instant solution, at least as far as its practical aspect is concerned. With a few drops of oil of orange we accomplish all that formerly demanded much preliminary psychic care, gas oxygen inhalations, and injections of novocain and quinine and urea hydrochloride before and after anesthesia. Are pleasant oders narcotic to the olfactory nerve? Is that explanation of their widespread and agelong use? Certain unpleasant oders are undoubtedly terrifying to animals, those of their enemies, for example. If pleasant odors are indeed sedative to the olfactory nerve, does that suffice to explain their extraordinary influence, at least when followed by ether, over the entire nervous system? Is there merely an association of ideas? The smell of ether is associated with the surgical knife in the minds of adults, and is terrifying to children from its irritating qualities. Perhaps the very familiarity and the harmlessness associated with the odors of flowers and fruit are sufficent to suggest powerfully to the subject that what he is about to undergo cannot be dangerous or even unfamiliar.

